# Gene Expression and Pathway Analysis of Effects of the CMAH Deactivation on Mouse Lung, Kidney and Heart

**DOI:** 10.1371/journal.pone.0107559

**Published:** 2014-09-17

**Authors:** Deug-Nam Kwon, Byung-Soo Chang, Jin-Hoi Kim

**Affiliations:** 1 Department of Animal Biotechnology, Konkuk University, Seoul, South Korea; 2 Department of Cosmetology, Hanseo University, Seosan, Chungnam, South Korea; Institute of Zoology, Chinese Academy of Sciences, China

## Abstract

**Background:**

N-glycolylneuraminic acid (Neu5Gc) is generated by hydroxylation of CMP-Neu5Ac to CMP-Neu5Gc, catalyzed by CMP-Neu5Ac hydroxylase (CMAH). However, humans lack this common mammalian cell surface molecule, Neu5Gc, due to inactivation of the CMAH gene during evolution. CMAH is one of several human-specific genes whose function has been lost by disruption or deletion of the coding frame. It has been suggested that CMAH inactivation has resulted in biochemical or physiological characteristics that have resulted in human-specific diseases.

**Methodology/Principal Findings:**

To identify differential gene expression profiles associated with the loss of Neu5Gc expression, we performed microarray analysis using Illumina MouseRef-8 v2 Expression BeadChip, using the main tissues (lung, kidney, and heart) from control mice and CMP-Neu5Ac hydroxylase (Cmah) gene knock-out mice, respectively. Out of a total of 25,697 genes, 204, 162, and 147 genes were found to be significantly modulated in the lung, kidney, and heart tissues of the Cmah null mouse, respectively. In this study, we examined the gene expression profiles, using three commercial pathway analysis software packages: Ingenuity Pathways Analysis, Kyoto Encyclopedia of Genes and Genomes analysis, and Pathway Studio. The gene ontology analysis revealed that the top 6 biological processes of these genes included protein metabolism and modification, signal transduction, lipid, fatty acid, and steroid metabolism, nucleoside, nucleotide and nucleic acid metabolism, immunity and defense, and carbohydrate metabolism. Gene interaction network analysis showed a common network that was common to the different tissues of the Cmah null mouse. However, the expression of most sialytransferase mRNAs of Hanganutziu-Deicher antigen, sialy-Tn antigen, Forssman antigen, and Tn antigen was significantly down-regulated in the liver tissue of Cmah null mice.

**Conclusions/Significance:**

Mice bearing a human-like deletion of the Cmah gene serve as an important model for the study of abnormal pathogenesis and/or metabolism caused by the evolutionary loss of Neu5Gc synthesis in humans.

## Introduction

Xenotransplantation using pig organs has the potential to solve the increasing shortage of donor organs available for allotransplantation [1]. Over the last two decades, there have been considerable advances in our understanding of the immunological and physiological hurdles to xenotransplantation. Many of the obstacles to xenotransplantion result from mismatches in receptor-ligand or enzyme-substrate interactions between the pig tissue and the recipient's blood and immune system [2]. The initial cause of failure for pig cardiac and renal xenografts is thought to be antibody-mediated injury to the endothelium, leading to the development of microvascular thrombosis. Factors contributing to the development of thrombotic microangiopathy include anti-non-Gal antibodies, natural killer cells or macrophage activity, and inherent coagulation dysregulation between pigs and primates [3]. To address these problems, several researchers have produced gene knockouts or transgenic animals to correct these mismatches. However, the combination of these modifications into an “idealized” transgenic animal has yet to be reported.

Neu5Gc is produced from Neu5Ac through enzymatic hydroxylation of the N-acetyl residue of free Neu5Ac, CMP-Neu5Ac, or glycoconjugate-linked Neu5Ac [4], [5]. Neu5Gc, also called Hanganutziu-Deicher (H-D) antigen, is expressed on the endothelial cell surface of all mammals, with the exception of humans, and is a target for non-Galα 1,3 Gal antibodies [6]. As consequence of inactivation of the CMP-Neu5Ac hydroxylase (Cmah) gene during evolution, humans have lost the ubiquitous mammalian cell surface molecule Neu5Gc [7], [8], [9]. Conversely, Neu5Gc produced by CMAH activity is one of the non-Gal xenoantigens of secondary importance to α-1, 3-galactosyltransferase (GGTA1), for pig-to-human xenotransplantation [10]. Similar to the galactose α1,3 galactose (α-Gal), Neu5Gc is immunogenic in humans as it is responsible for the expression of Neu5Gc, a key non-Gal antigen [11]. Very recently, we have developed a biallelic CMAH knock-out in pigs [12]. However, these data raise the possibility of an alternate pathway in metabolism and the immune system, which might contribute to acute immune rejection of xenografts.

The silencing of the CMAH gene expression resulted in a number of genetic and biochemical changes to the biosynthesis of sialic acids, which may have contributed to several unique aspects of human biology in health and disease [13], [14]. Cmah null mice show Nue5Ac accumulation, a characteristic present in humans. The Cmah null mice also exhibit many of the problems common in humans, including diminished acoustic sensitivity and startle response threshold, which resulted in hearing loss and delays to skin healing [15], [16]. Recently, microarray analysis has been shown to be a powerful tool for the analysis of gene expression and is particularly suited for the identification of transcription factor target genes. In addition, network-assisted analysis of DNA chip data is an emerging area in which network-related approaches are developed and utilized in order to study human diseases or traits. To identify the transcriptional alterations caused by the human-specific loss of Neu5Gc, we examined expression profiling of the main organs, which included the lung, kidney, and heart harvested from wild type (WT) and Cmah null mice. Here, we report the use of network-assisted transcript profiling to various diseases and discuss the options relating to practical applications.

## Materials and Methods

### Animal ethics

All animal experiments were approved and performed under the guidelines of the Konkuk University Animal Care and Experimentation Community [IACUC approval number: KU12045]. Cmah <tm1Ykoz> knockout mice were kindly provided by RIKEN (Japan). All lines were maintained on a congenic C57Bl/6J background. The mice were allowed to eat and drink ad libitum and were fed with standard mouse chow (Cargill Agri Purina, Inc., Seongnam-Si, Korea). Twelve weeks old wild type and Cmah null male mouse in this study were used.

### Immunohistochemistry (IHC)

For IHC, the tissues were fixed in neutral buffer with 10% formalin and then embedded on slides. Endogenous peroxidase activity was blocked using 3% hydrogen peroxide. The samples were then pretreated with Borg Decloaker, and blocked in background Sniper solution. After washing, the samples were incubated with specific primary antibodies for CMAH (Santa Cruz; Texas, USA, 1∶100) and Neu5Gc (Sialix; San Diego, CA, USA; 1∶200) at 4°C overnight. After the incubation, the samples were washed and incubated with horseradish peroxidase-conjugated secondary antibody. Samples were then stained with ImmPACT™ DAB peroxidase substrate (Vector Laboratories; CA, USA) to visualize the signal. Samples were also stained with Hematoxylin QS to provide background information for reference. The samples were mounted using VECTORSHIELD HardSet mounting medium (Vector Laboratories; CA, USA) and observed using fluorescence microscopy (Olympus; Japan).

### Microarray analysis

WT and Cmah null mice with same age (12 weeks) and genetic background (C57BL/6J) were used (n = 3 per each group) for microarray analysis. Total RNA was extracted and purified from the lung, kidney, and heart of WT and Cmah null mice using RNeasy columns (Qiagen; Valencia, CA, USA) according to the manufacturer's protocol. The RNA quality was verified using an Agilent Bioanalyzer 2100 (Agilent Technologies; Palo Alto, CA, USA) using the RNA 6000 Pico Assay. Generation of double-stranded cDNA, preparation and labeling of cRNA, hybridization to Mouse Ref-8 v2.0 Expression BeadChip (Illumina, Inc.; San Diego, CA, USA), washing, and scanning were all performed according to the standard Illumina protocol. Arrays were scanned using the Illumina Bead Array Reader Confocal Scanner. Microarray data have been deposited in NCBI's Gene Expression Omnibus and are accessible through GEO Series accession number GSE59964 (Gene expression profile of the Cmah gene depletion on mouse liver, lung, kidney, and heart).

### Raw data preparation and statistical analysis

The quality of hybridization and overall chip performance were monitored by visual inspection of both internal quality control checks and the raw scanned data. The raw data were extracted using the software provided by the manufacturer (Illumina GenomeStudio v2009.2 [Gene Expression Module v1.5.4]). Array data were filtered by a detection p-value < 0.05 in at least 50% of the samples. Selected gene signal values were logarithm-transformed and normalized using the quantile method [17]. Comparative analysis between the wild-type group and Cmah null mice group was carried out based on fold-change in expression levels.

### Gene ontology (GO) analysis

GO analysis of the significant probe list was performed using PANTHER (http://www.pantherdb.org/), using text files containing the Gene ID list and accession numbers of the Illumina probe ID. All data analysis and visualization of differentially expressed genes were conducted using R 2.4.1 (www.r-project.org). In addition, the DAVID Functional Annotation Bioinformatics Microarray Analysis tools (http://david.abcc.ncifcrf.gov/) were used to study the biological function of the regulated genes [18].

### Kyoto Encyclopedia of Genes and Genomes (KEGG) pathway analysis

KEGG is a collection of online databases dealing with genomes, enzymatic pathways, and biological chemicals [19]. The PATHWAY database records networks of molecular interactions in the cell that includes organism-specific network maps (http://www.genome.jp/kegg/). A total of 9 pathways, involving 49 genes, were collected from KEGG.

### Interactome/network analysis of differentially expressed genes

Significantly affected or differentially expressed genes were subjected to a comprehensive search to identify their biological functions. Gene interaction networks, bio functions, and pathway analysis were generated by Ingenuity Pathway Analysis (IPA) (Ingenuity Systems; Mountain View, CA, USA), which assists with microarray data interpretation via grouping of differentially expressed genes into known functions, pathways, and networks primarily based on human and rodent studies. The identified genes were mapped to genetic networks available from the Ingenuity database and were then ranked by score. The significance was set at a p-value of 0.05.

### Pathway Studio analysis

To identify molecular pathways, we arranged the data by relation type, using Pathway Studio 9.0 software (Ariadne Genomics; Rockville, MD, USA). This program integrates relevant information among the imported genes, consequently allowing identification of biological pathways, gene regulation networks, and protein interaction maps.

### Quantitative real-time polymerase chain reaction (RT-qPCR)

The total RNAs obtained from each tissues (lung, kidney, heart, and liver) used for microarray analysis was reverse-transcribed with the QuantiTect Reverse Transcription Kit (Qiagen; Valencia, CA, USA), according to the manufacturer's recommendations. To assess gene expression, quantitative real-time polymerase chain reaction (RT-qPCR) was performed on an ABI ViiA™ 7 system (Applied Biosystems; Foster City, CA, USA) with SYBR Green as the fluorescence detection method (Bio-Rad; Hercules, CA, USA). Specific primer sets for RT-qPCR were listed in Table S1 and S2. The mouse glyceraldehyde-3-phosphate dehydrogenase (Gadph) gene was used as an internal control to normalize the RT-qPCR efficiency and for quantification of gene expression. After normalization with Gadph expression, we compared the relative expression of each mRNA sample in the Cmah null mice with those of the controls. The RT-qPCR was performed in triplicate for each sample.

## Results

### Disruption of the Cmah gene completely abolishes Neu5Gc production

The production of Cmah null mice has been described previously [15], [16]. The Cmah null mouse was confirmed by PCR and the lack of CMAH protein expression was confirmed by western blotting analysis (data not shown). When Cmah null mice from Cmah^+/−^ × Cmah^+/−^ crosses were examined and weighed, homozygous Cmah null mice were indistinguishable from their wild type or heterozygous littermates in all respects.

**Figure 1:**
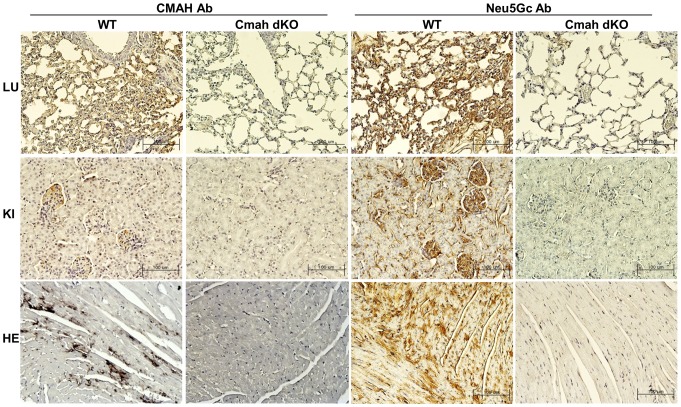
Immunohistochemistry in Cmah-dKO mouse-derived tissues (lung, kidney, and heart) for detection of CMAH and Neu5Gc. WT mice expressed both Cmah and Neu5Gc epitopes. Cmah-dKO mice were completely deficient for both CMAH and Neu5Gc epitopes in the lung, kidney, and heart tissues. Bar: 100 µm. LU; lung, KI; kidney, HE; heart.

IHC was used to determine expression patterns for Cmah and Neu5Gc in the lung, kidney, and heart tissues of control and Cmah null mice at 12 weeks of age, using specific antibodies for CMAH and Neu5Gc. As shown in Figure 1, both the CMAH protein and Neu5Gc epitope were highly expressed in the control mice tissues, whereas the Cmah null mice were completely deficient for both Cmah and Neu5Gc in the lung, kidney, and heart. These observations suggested that the lung, kidney and heart tissues of the Cmah null mouse may contain possible molecular targets associated with xenoantigens relevant to animal-to-human xenotransplantation or human species-specific metabolic syndrome.

### Cmah null mice show reduced sialyltransferase-related gene expression

**Figure 2:**
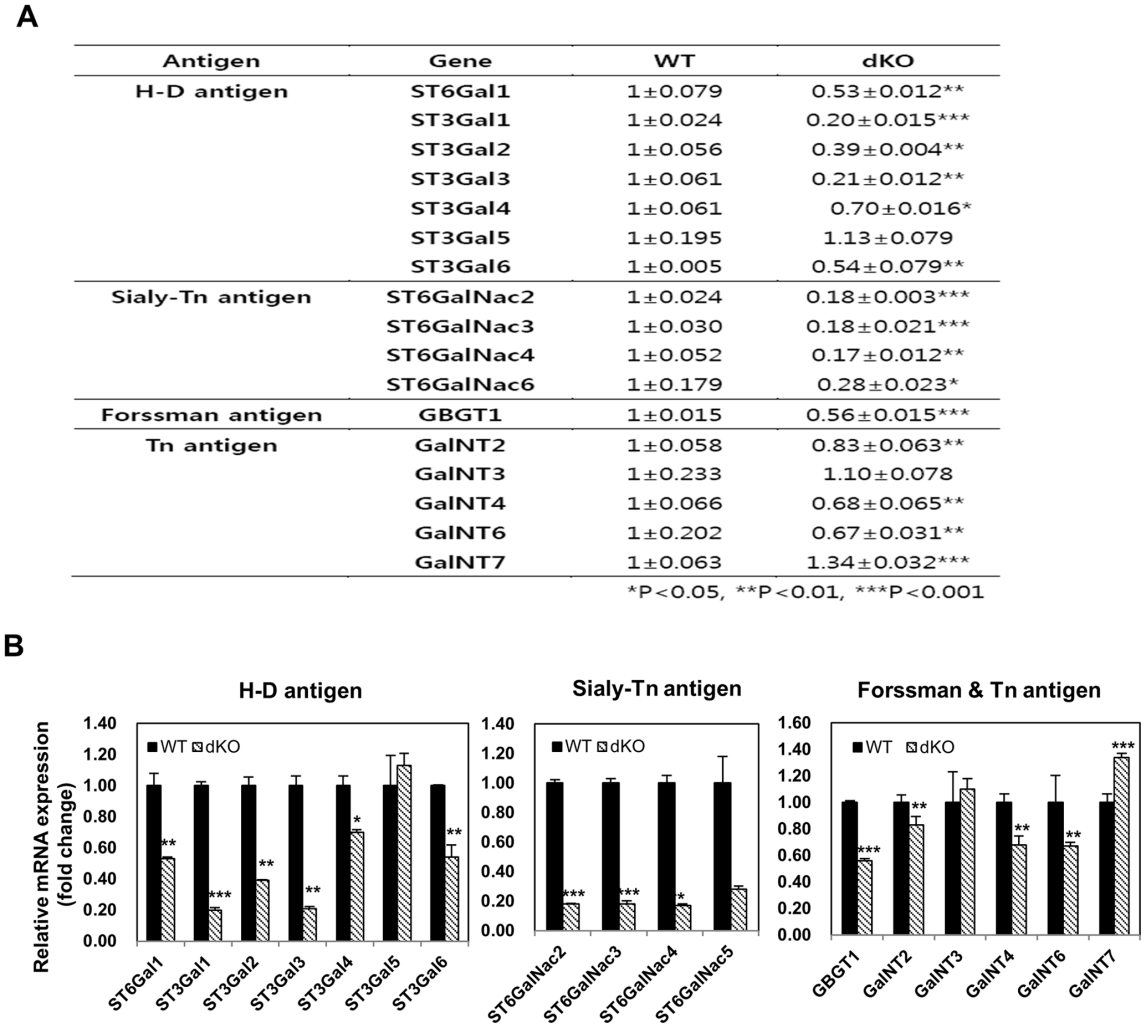
Sialyltransferase gene expression levels in WT and Cmah-dKO mice. A. Comparison of sialyltransferase gene expression in the liver of WT and Cmah-dKO mice by RT-qPCR. B. RT-qPCR analysis in liver of WT and Cmah-dKO mice. All RT-qPCR were conducted in triplicate and normalized with mouse *Gadph* gene expression. The data were presented as mean ± SD. *P<0.05, **P<0.01, ***P<0.001.

Neu5Gc, also called the H-D antigen, is produced by the CMAH enzyme and is one of the non-Gal xenoantigens for pig-to-human xenotransplantation [10]. Therefore, we examined the sialyltransferase (ST3Gal1–4 and ST6Gal1) expression pattern in the livers of control and Cmah null mice. Gadph gene expression was simultaneously assayed as an endogenous invariant control for data normalization. Compared with the H-D antigen gene expression in the control mouse (1±0.079; 1±0.024; 1±0.056; 1±0.061; 1±0.061; 1±0.005), RT-qPCR analysis showed that Cmah null mice had low ST6Gal1 (0.53±0.012), ST3Gal1 (0.20±0.015), ST3Gal2 (0.39±0.004), ST3Gal3 (0.21±0.012), ST3Gal4 (0.70±0.0016), and ST3Gal6 (0.54±0.079) expression levels, whereas the ST3Gal5 expression level was slightly elevated (1.13±0.079) (Figure 2). Interestingly, the H-D antigen mRNA expression in Cmah null mice was not completely lost, suggesting that the H-D antigen mRNA expression may be compensated when one gene does not function for some reason. Since ST3GAL1, 3, and 4 catalyze the addition of Neu5Ac to the nonreducing terminal of the galactose (Gal) residue of glycans, our observations indicate that down-regulation of ST3GAL1, 3, and 4 and ST6GAL1 expression in Cmah null mice may reduce cytotoxic T cell numbers and therefore attenuate resistance to pathogens and tumorigenic lesions.

Expression levels of the Sialy-Tn antigen-related genes ST6GalNac2 (0.018±0.003), ST6GalNac3 (0.018±0.021), ST6GalNac4 (0.017±0.012), and ST6GalNac6 (0.28±0.023), were significantly lower in the Cmah null mice than in the control mice (1±0.024, 1±0.030, 1±0.052, and 1±0.179, respectively; Figure. 2A). Expression levels of GBGT1 (0.56±0.015) for the Forssman antigen, and of GalNT2 (0.83±0.063), GalNT4 (0.68±0.065), and GalNT6 (0.67±0.031) for the Tn antigen in Cmah null mice were also significantly lower in comparison to those of the control mice (1±0.058, 1±0.066, and 1±0.202, respectively). However, GalNT7 expression for the Tn antigen, unlike other family members, was significantly increased. Considering that the GalNT7 enzyme shows exclusive specificity for partially GalNAc-glycosylated acceptor substrates and shows no activity with non-glycosylated peptides, increased GalNT7 expression in the Cmah null mouse may promote the initiation of the O-glycosylation step.

We next analyzed the binding of natural xenoreactive antibodies in human serum using control (WT), Cmah heterozygote (mKO), and Cmah homozygote (Cmah dKO or Cmah null)) mice. As shown in Figure S1, most of the serum samples from healthy volunteers with different blood groups (A, B, O, and AB) showed the presence of naturally occurring IgM and IgG antibodies that were bound to thymocytes from WT, Cmah-mKO, Cmah-dKO mice. Of note, IgG binding to Cmah dKO mouse-derived thymocytes was significantly reduced in all tested blood groups compared to WT- or Cmah mKO-derived thymocytes. Whereas, there was no significant IgM antibody in the A, O, and AB blood types.

### Microarray analysis of gene expression profiles in the lung, kidney, and heart of Cmah null mice

**Figure 3:**
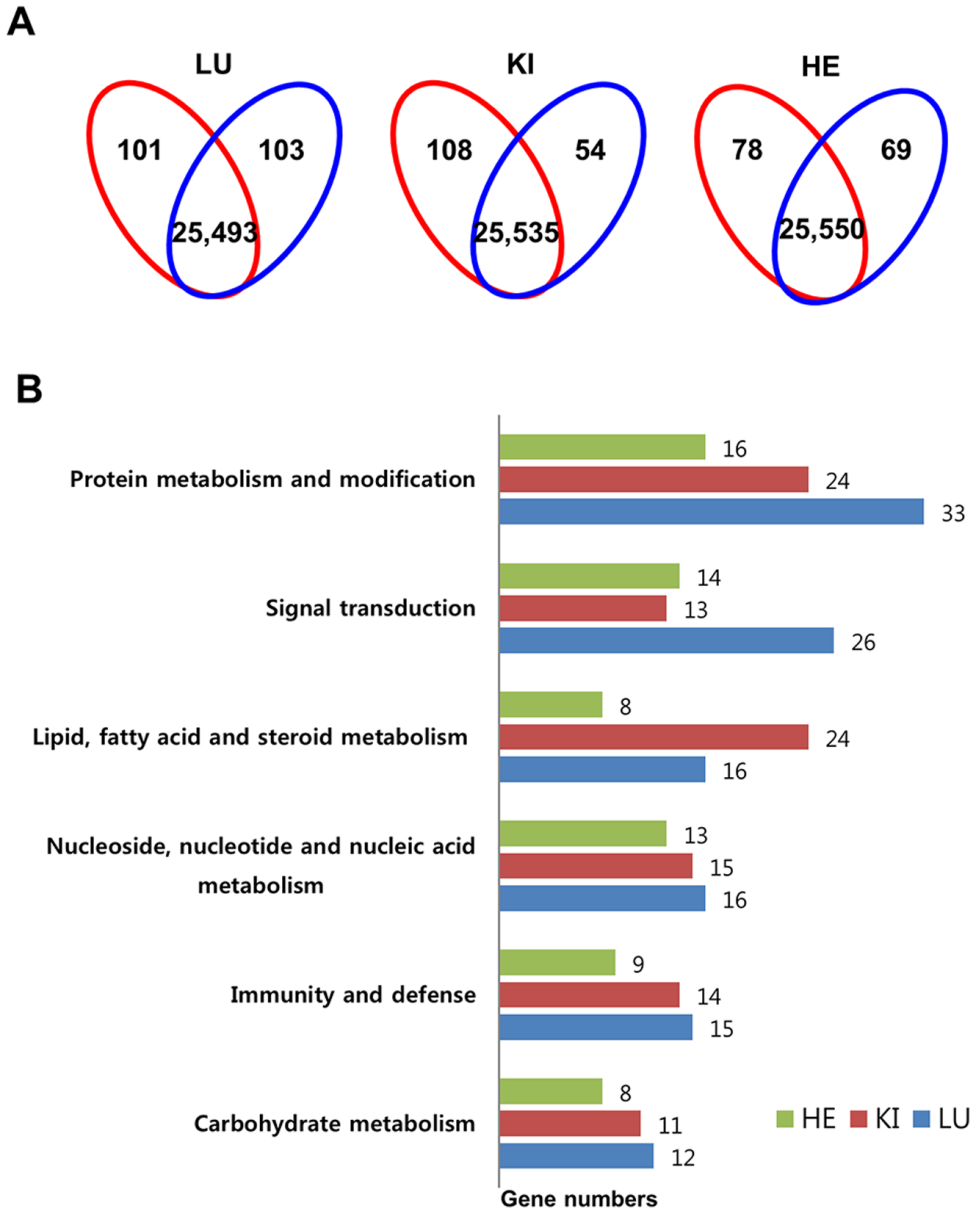
Gene expression profile in the lung, kidney, and heart of Cmah null mice. A. Venn diagram showing differential expression of genes in the lung, kidney, and heart of Cmah null mice. Numbers in red and blue Venn diagram present up- and down- regulated genes, respectively. LU, lung; KI, kidney; HE, heart. B. The differentially up- or down-regulated genes were classified according to biological process in the lung, kidney, and heart of Cmah-dKO mice. LU, lung; KI, kidney; HE, heart. X axis of bar graph indicates the gene numbers.

Following microarray analysis using the Illumina MouseRef-8 v2 Expression BeadChip (composed of 25,697 filtered probe sets) using the main tissues (lung, kidney, and heart) from the control and Cmah null mouse groups (n = 3, respectively), all hybridization spots on the image were quantified. The fluorescence intensity data were converted into log_10_ values. Genes with significantly different expression levels (P<0.05) in the lung, kidney, and heart from the Cmah null mouse, relative to the control mouse, were extracted for further analysis. The results showed that 204, 162, and 147 genes were differentially expressed, with a more than 1.5-fold change in the lung, kidney, and heart of Cmah null mice when compared with age- and sex-matched control mice, respectively (Figure 3A). In the lung, 101 genes were up-regulated and 103 genes were down-regulated (File S1). In the kidney, 108 genes were up-regulated and 54 genes were down-regulated (File S2). In the heart, 78 genes were up-regulated and 69 genes were down-regulated (File S3). Even though 2 up-regulated genes (Entpd4 and Dex3y) and 4 down-regulated genes (Nr1d1, Sybl1, LOC10047427, and Cfd) overlapped in all tissues (Table S3), overall, the overlapping between 2 different organs (lung vs. heart, kidney vs. heart, and lung vs. kidney) and 3 different organs (lung vs. kidney vs. heart) was minimal.

### GO enrichment, KEGG, and IPA pathway analysis of differentially expressed genes

To understand their biological roles, the genes with significant changes in expression detected in Cmah null mice by microarray analysis were assigned to establish GO classification categories using the PANTHER and DAVID tools. There are 3 ontologies in GO: cellular component, molecular function, and biological process [20]. In GO classification, the top 6 enriched biological processes from the GO analysis were categorized according to their functional role, as shown in Table 1 and Files S1, S2, and S3. These biological groups covered 5 subcategories (protein biosynthesis, protein complex assembly, protein folding, protein modification, and proteolysis) for protein metabolism and modification, 3 subcategories (cell communication, cell surface receptor-mediated signal transduction, and intracellular signaling cascade) for signal transduction, 4 subcategories (fatty acid metabolism, lipid and fatty acid transport, lipid metabolism, and steroid metabolism) for lipid, fatty acid, and steroid metabolism, 10 subcategories (antioxidation and free radical removal, B-cell- and antibody-mediated immunity, blood clotting, complement-mediated immunity, cytokine/chemokine-mediated immunity, detoxification, interferon-mediated immunity, macrophage-mediated immunity, stress response, and T-cell-mediated immunity) for immunity and defense, 6 subcategories (carbohydrate transport, gluconeogenesis, glycogen metabolism, glycolysis, pentose-phosphate shunt, and tricarboxylic acid pathway) for carbohydrate metabolism, and 3 subcategories (mRNA transcription, purine metabolism, pyrimidine metabolism) for nucleoside, nucleotide, and nucleic acid metabolism. Although expression profiles were completely different for each tissue of the Cmah null mice, some genes were shared between tissues in a biological group (Fkbp5, Cfd, Dbp, and Entpd4).

**Table 1:** Top 6 biological processes overrepresented in the lung, kidney, and heart of Cmah null mice.

Biological group	Functional role	Lung	Kidney	Heart	Biological group	Functional role	Lung	Kidney	Heart
Protein metabolism and modification (P = 1.26E-07 in LU, P = 0.019 in KI, P = 0.042 in HE)	Protein biosynthesis	Rps3(−1.58)	Rpl23(−1.62), Eif4ebp2(−1.56)	Rpl23(−1.71)	Immunity and defense (P = 0.022 in LU, P = 6.44E-06 in KI, P = 0.045 in HE)	Cytokine/chemokine mediated immunity	Ccl11(−1.52), Gab3(1.59)		Tnfaip2(−1.97), Ccl9(1.67)
	Protein complex assembly	Hspa1a(1.65)		Hspa8(1.55)		Detoxification		Abcc3(−3.41), Gsta2(−1.76)	Gsta3(1.89), Gpx3(−1.60)
	Protein folding	Fkbp5(−1.94), Dnajb6(1.52), Hspb1(1.70), etc.	Fkbp5(1.53)	Hspa8(1.55), Eif4ebp2(−1.56), Fkbp5(2.05)		Interferon-mediated immunity	Gbp6(1.54), Gbp10(1.56), Gbp3(1.75), etc.	Oasl2(1.57), Ifit3(1.65), Irf7(1.92)	Gbp2(1.56)
	Protein modification	A4galt(−1.66), Mapk9(1.63), Bmx(1.86), etc.	Wnk1(−1.66), Pdk4(−1.66), Cml2(1.57), Cml3(1.61)	Wnk1(−1.64), Map3k6(1.53), Man2a1(1.69), etc.		Macrophage-mediated immunity	Sdc4(−1.55), Gbp6(1.54), Gab3(1.59), etc.	Asgr1(1.56), Fcgr4(1.80)	Scara3(−2.01), Tnfaip2(−1.97), Gbp2(1.56)
	Proteolysis	Ela2a(−12.87), Try4(−3.12), Cfd(−2.67), Ctrl(−2.61), Cpb1(−2.31), etc.	Cfd(−3.01), Itih1(1.71), Serpina1e(4.72), etc.	Cfd(−4.45), Mmp14(−1.85), Usp2(1.69), Ela2a(4.32), etc.		Stress response	U4606(−4.81), Plunc(−2.61), Hspb1(1.70), Bmx(1.86), etc.	Hp(2.19)	Gpx3(−1.60), Ier3(1.51), Lyz2(1.67), etc.
Signal transduction (P = 0.023 in LU, P = 0.049 in KI, P = 0.043 in HE)	Cell communication	Adam23(−1.76), Per1(−1.52), Cap1(2.77), etc.	Foxq1(−2.17), Per1(−1.58), Foxa3(1.53), Cap1(2.31), etc.	Chad(−2.11), Itgb6(−1.84), Igf2(−1.77), Per2(3.47), etc.		T-cell mediated immunity	Fkbp5(−1.94), Gab3(1.59), Bmx(1.86), etc.	Fkbp5(1.53), Azgp1(2.09)	Sqstm1(1.51), Ab1(1.53), Fkbp5(2.05), etc.
	Cell surface receptor mediated signal transduction	Tff2(−3.65), Cd8b1(−1.65), Edn1(1.64), Gpr182(2.07), etc.	Prlr(−2.41), Tob2(−1.53), Foxa3(1.53), Lrg1(1.66), etc.	Rrad(−1.69), P2ry1(1.66), Ccl9(1.67), etc.	Carbohydrate metabolism (P = 0.0003 in LU, P = 0.007 in KI, P = 0.0003 in HE)	Carbohydrate transport	Slc35b1(1.59)	Slc2a2(−1.52), Pck1(1.55)	Adipoq(−1.64)
	Intracellular signaling cascade	Fkbp5(−1.94), Mapk9(1.63), Bmx(1.86), etc.	Prlr(−2.41), Wnk1(−1.66), Fkbp5(1.53), Cap1(2.31), etc.	Wnk1(−1.64), Pik3r1(1.87), Fkbp5(2.05), etc.		Gluconeogenesis			Adipoq(−1.64)
Lipid, fatty acid and steroid metabolism (P = 1.61E-05 in LU, P = 2.70E-06 in KI, P = 4.74E-05 in HE)	Fatty acid metabolism	Cyp2a5(−2.58), Dgat2(−1.68), Acoxl(−1.53), etc.	Cyp4a14(−2.33), Cyp2a5(1.66), Cyp4a12a(3.28), etc.	Scd1(−2.41), Scd4(−1.97), Adipoq(−1.64), Ptgds(2.01)		Glycogen metabolism	Amy2(−2.63)		
	Lipid and fatty acid transport		Apoc3(1.74), Apoa2(1.95), Apoc1(2.09)			Glycolysis	6430537H07Rik(−1.52)		Pfkm(1.68), Gck(2.68)
	Lipid metabolism	Pnlip(−5.43), Clps(−4.93), Pnliprp1(−3.07), Cel(−2.36), etc.	Lip1(−1.67)	Pnlip(1.74), Clps(1.76), Ptgds(2.01)		Pentose-phosphate shunt	Tkt(1.81)		
	Steroid metabolism		Ugt1a2(−4.03), Cyp27b1(−2.12), Rdh7(1.58), etc.	Gpx3(−1.60), Ela1(1.63)		Tricarboxylic acid pathway	Mdh1b(−1.51)		Idh1(1.59)
Immunity and defense (P = 0.022 in LU, P = 6.44E-06 in KI, P = 0.045 in HE)	Antioxidation and free radical removal		Sepp1(1.87)	Gpx3(−1.60)	Nucleoside, nucleotide and nucleic acid metabolism (P = 0.0002 in LU, P = 4.43E-05 in KI, P = 2.15E-07 in HE)	mRNA transcription	Dbp(−4.22), Nr1d1(−2.26), Fos(−2.03), Per1(−1.52), etc.	Foxq1(−2.17), Dbp(−1.66), Per2(1.51), Foxa3(1.53), etc.	Egr1(−2.75), Fos(−1.56), Dbp(2.74), Per2(3.47), etc.
	B-cell- and antibody-mediated immunity	Vpreb3(−1.70), Cd69(−1.51), Gab3(1.59), etc.	Fcgr4(1.80)			Purine metabolism	Entpd4(1.80)	Entpd4(2.12)	Entpd4(1.65)
	Blood clotting	Anxa8(−1.55)	F2(1.56), Asgr1(1.56), Fga(1.87)	Angptl4(−1.94), Itgb6(−1.84), Vwf(−1.54)		Pyrimidine metabolism	Nme5(−1.78)	Upp2(1.59)	
	Complement-mediated immunity	Cfd(−2.67)	Cfd(−3.01), C4b(1.89), C3(1.79), etc.	Cfd(−4.45)					

The results generated using the PANTHER classification system is shown in Figure 3B and Table 1. Each gene was assigned to one or more biological groups, according to the function of its proteins. The classification was based on 31 biological groups; the 6 groups that contained the majority of the differentially expressed genes were protein metabolism and modification (33, 24, and 16 genes in the lung, kidney, and heart, respectively), signal transduction (26, 13, and 14 genes in the lung, kidney, and heart), lipid, fatty acid, and steroid metabolism (16, 24, and 8 genes in the lung, kidney, and heart), nucleoside, nucleotide, and nucleic acid metabolism (16, 15, and 13 genes in the lung, kidney, and heart), immunity and defense (15, 14, and 9 genes in the lung, kidney, and heart), and carbohydrate metabolism (12, 11, and 8 genes in the lung, kidney, and heart) (Figure 3B). In addition, GO classification by molecular function showed that differentially expressed genes were assigned 29 different functions (viral protein, cell junction protein, isomerase, chaperone, lyase, ion channel, synthase/synthetase, cell adhesion molecule, extracellular matrix, select calcium binding protein, defense/immunity protein, phosphatase, transfer/carrier protein, membrane traffic protein, protease, ligase, cytoskeletal protein, signaling molecule, transporter, hydrolase, oxidoreductase, miscellaneous function, kinase, transferase, receptor, select regulatory molecule, transcription factor, nucleic acid binding, and molecular function unclassified), while 42 (24 down-regulated genes and 18 up-regulated genes), 23 (5 down-regulated genes and 18 up-regulated genes), and 28 (15 down-regulated genes and 13 up-regulated genes) genes had multiple functions in the lung, kidney, and heart, respectively (Files S1, S2, and S3). The GO classification by cellular component using the DAVID tool showed that 34 differentially expressed genes were mapped to cell fraction, membrane fraction, plasma membrane, etc.; 32 genes mapped to extracellular region, insoluble fraction, organelle membrane, etc.; and 36 genes mapped to extracellular matrix, cytoplasmic vesicle, organelle lumen, etc. in the lung, kidney, and heart, respectively (data not shown).

The molecular pathways associated with the differentially expressed genes from the lung, kidney, and heart of Cmah null mice were identified using KEGG pathway analysis. KEGG pathways are manually drawn maps representing well-known molecular interaction and reaction networks. Differentially expressed genes from the lung, kidney, and heart of Cmah null mice were associated with the following pathways (P<0.05): for the lung, retinol metabolism and glycerolipid metabolism; for the kidney, drug metabolism, arachidonic acid metabolism, complement and coagulation cascades, retinol metabolism, fatty acid metabolism, and circadian rhythm; for the heart, focal adhesion (Table 2). These data indicate that deletion of the Cmah gene leads to both up- and down-regulation of gene expression in the lung, kidney, and heart tissues, which may regulate the metabolism and signaling pathway within these tissues.

**Table 2:** KEGG pathways significantly associated with genes altered in the lung, kidney, and heart of Cmah null mice.

Tissues	Term	Count	P value	Genes
Lung	Retinol metabolism	6	0.0006147	Alah1a1, Dgat2, Cyp26b1, Cyp2a5, Adh7, Aldh1a7
	Glycerolipid metabolism	4	0.0121528	Pnlip, Cel, Pnliprp1, Dgat2
Kidney	Drug metabolism	9	0.0000009	Cyp2d9, Gsta2, Fmo5, Ugt2b36, Adh1, Ugt1a2, Cyp2a5, Cyp2d26, Cyp2e1
	Arachidonic acid metabolism	5	0.0107399	Cyp4a12b, Cyp2j13, Cyp4a12a, Cyp2e1, Cyp4a14
	Complement and coagulation cascades	10	0.0000001	Mbl1, Fga, Serpina1b, C3, Serpina1a, C4b, Serpina1d, F2, Serpina1e, Cfd
	Retinol metabolism	7	0.0000671	Cyp4a12b, Ugt2b36, Cyp4a12a, Adh1, Ugt1a2, Cyp2a5, Cyp4a14
	Fatty acid metabolism	4	0.0112964	Cyp4a12b, Cyp4a12a, Adh1, Cyp4a14
	Circadian rhythm	3	0.0077886	Nr1d1, Per2, Per1
Heart	Focal adhesion	8	0.0010197	Actb, Vwf, Myl7, Col4a1, Itgb6, Col1a1, Pik3r1, Chad

**Figure 4:**
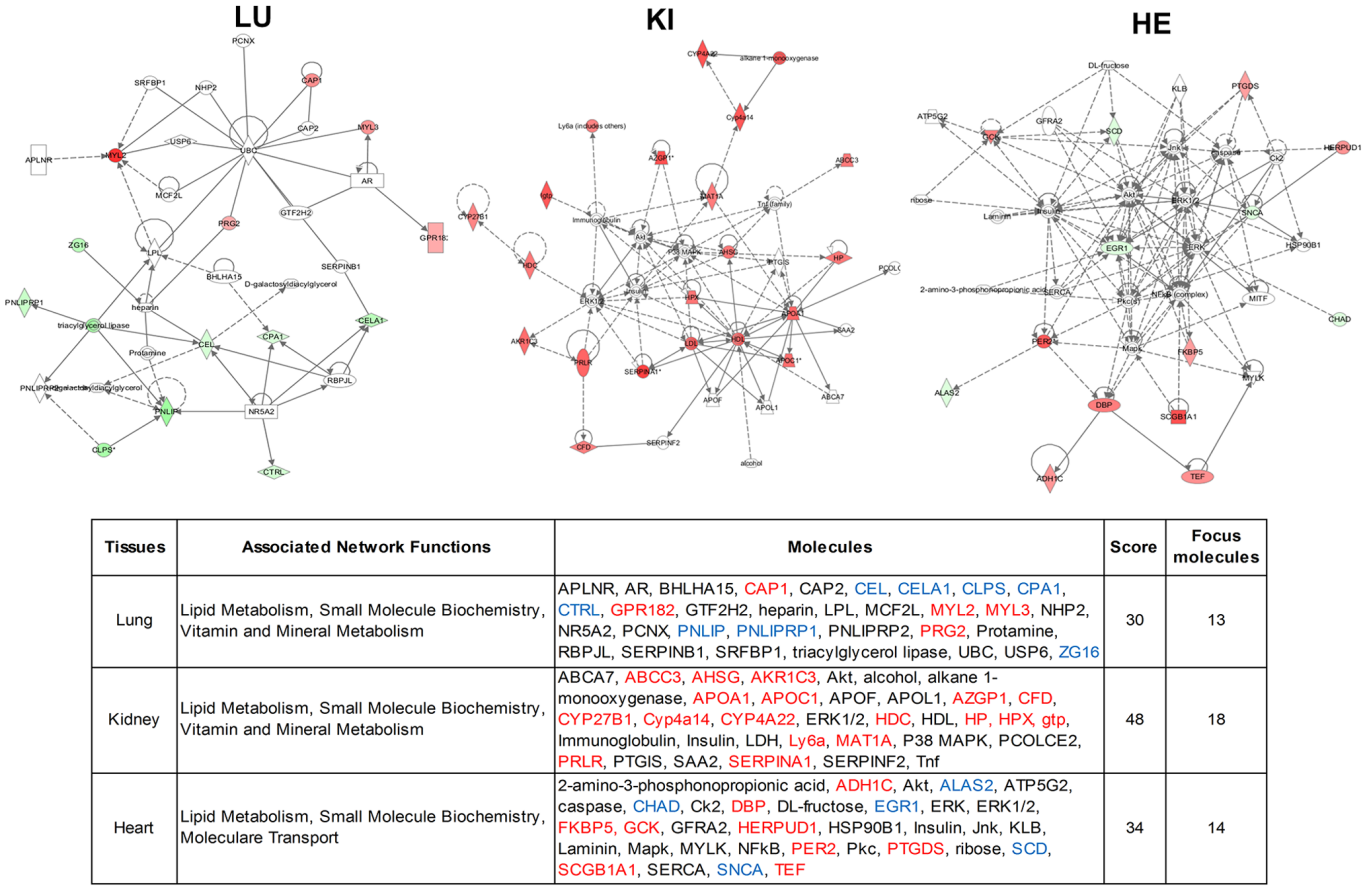
Networks predicted by Ingenuity Pathway Analysis in the Cmah-dKO mice. Upper: The common network identified was lipid metabolism and small-molecule biochemistry in the lung (LU), kidney (KI), and heart (HE), respectively. The network is displayed graphically as nodes (genes). The node color intensity indicates the expression of genes, with red representing up-regulation and green representing down-regulation. Solid lines and dotted lines indicate direct relationship and indirect relationships, respectively. Bottom: The table includes the molecules, score, and focus molecules in identified common networks (lipid metabolism and small molecule biochemistry) from each tissue by IPA (score 30, 13 focus molecules in LU; score 48, 18 focus molecules in KI; score 34, 14 focus molecules in HE). Red and blue denote up-regulated and down-regulated genes among the differentially expressed genes.

We then performed IPA, which identified 3 gene interaction networks identified in the Cmah null tissues from the uploaded gene lists, based on the literature contained in the IPA knowledge base (P<0.05) (Table S4). The analysis revealed one network associated with lipid metabolism and small molecular biochemistry that was common to all tissue types (Figure 4). The network was merged and clustered around several central genes, including the up- and down-regulated genes identified from the microarray data (40.63% of the total nodes in the lung, 51.43% of the total nodes in the kidney, and 41.18% of the total nodes in the heart). This network contained 5 up-regulated genes (Cap1, Gpr182, Myl2, Myl3, and Prg2) and 8 down-regulated genes (Cel, Cela1, Clps, Cpa1, Ctrl, Plip, Pnliprp1, and Zg16) in the lung, whereas all the genes (Abcc3, Ahsg, Akr1c3, Apoa1, Apoc1, Azgp1, Cfd, Cyp27b1, Cyp4a14, Cyp4a22, Hdc, Hp, Hpx, Gtp, Ly6a, Mat1a, Prlr, and Serpina1) were up-regulated in the kidney. Similarly, the network in the heart was composed of 9 up-regulated genes (Adh1c, Dbp, Fkbp5, Gck, Herpud1, Per2, Ptgds, Scgb1a1, and Tef) and 5 down-regulated genes (Alas2, Chad, Egr1, Scd, and Snca).

IPA pathway analysis identified putative disease and disorders in lung, kidney, and heart of Cmah null mouse, respectively. These are as follows: inflammatory response, cardiovascular disease, developmental disorder, and skeletal and muscular diseases for lung, gastrointestinal disease, nutrition disease, and cancer for kidney, neurological disease, cancer, cardiovascular disease, and skeletal and muscular diseases for heart (Table 3). Taken together, the predicted interacting molecular networks in the Cmah null tissues from the IPA suggest that loss of Neu5Gc production has affected the signaling pathways that involved in human disease and disorders. Therefore, this result provides further information for more detailed evaluation of the potential effects of the loss of function of CMAH.

**Table 3:** Disease and disorders predicted by Ingenuity Pathway Analysis in lung, kidney, and heart of Cmah null mice.

Tissues	Lung (molecules)	Kidney (molecules)	Heart (molecules)
Disease and disorders	Inflammatory response (10)	Gastrointestinal disease (13)	Neurological disease (11)
	Cardiovascular disease (3)	Nutrition disease (7)	Cancer (9)
	Developmental disorder (5)	Cancer (21)	Cardiovascular disease (3)
	Skeletal and muscular diseases (6)		Skeletal and muscular diseases (12)

### RT-qPCR validation

**Figure 5:**
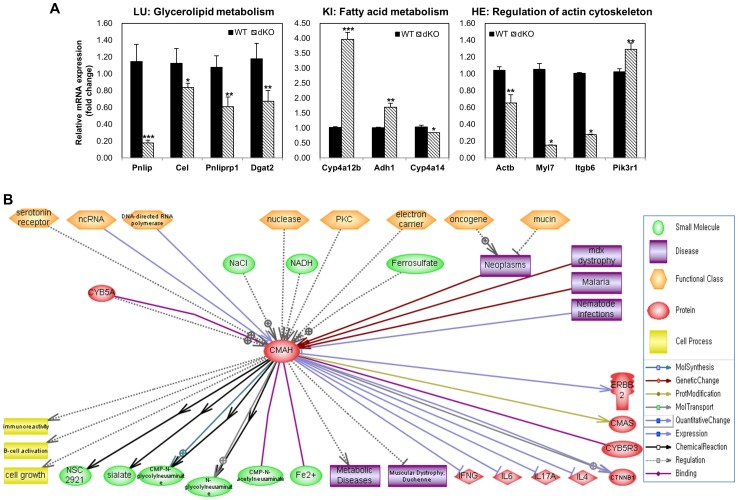
Schematic representation of biological networks was constructed by CMAH function. A. Validation of microarray gene expression by RT-qPCR. Gene expression levels of selected genes were determined by RT-qPCR using WT and Cmah-dKO derived lung, kidney, and heart tissues. Measurements were performed in triplicate, after which the calculated mean expression level was corrected for Gadph gene expression levels. Error bars indicate standard deviations. B. Functional correlation and interaction of both the signaling proteins and Cmah gene were reconstituted into a network model using the Pathway Studio software (Ariadne Genomics Inc.; Rockville, MD, USA). All genes are shown by their gene symbols. Direct and indirect regulation is indicated by colored lines and dotted gray lines, respectively.

The microarray expression data were validated by RT-qPCR analysis using genes selected for analysis of KEGG pathway in the lung, kidney, and heart of Cmah null mice. As shown in Figure 5A, the expression levels of Pnlip (pancreatic lipase), Cel (carboxyl ester lipase), Pnliprp1 (pancreatic lipase related protein 1), and Dgat2 (diacylglycerol O-acyltransferase 2) for glycerolipid metabolism in the lung, Cyp4a12b (cytochrome P450, family 4, subfamily a, polypeptide 12B), Adh1 (alcohol dehydrogenase 1), and Cyp4a14 (cytochrome P450, family 4, subfamily a, polypeptide 14) for fatty acid metabolism in the kidney, Actb (actin, beta), Myl7 (myosin, light chain 7), Itgb6 (integrin beta 6), and Pik3r1 (phosphatidylinositol 3-kinase, regulatory subunit, polypeptide 1) for regulation of actin cytoskeleton in the heart corresponded to the microarray results. This shows that the data sets obtained from the microarray analysis accurately reflect the differential gene expression in the lung, kidney, and heart between the control and Cmah null mice (Figure 5A).

### Putative Cmah interaction network analysis

Heterogeneous high-throughput biological data has become readily available for a number of diseases. However, the amount of data points generated by such experiments does not facilitate manual integration of the information, which is required to design the most optimal therapy for a disease. In this study, we examined a novel computational workflow for designing a therapy strategy using the Pathway Studio Software. Using subnetwork enrichment analysis (Figure 5B), we identified eight CMAH downstream-localized proteins (ERBB2, CMAS, CYB5R3, CTNNB1, IL4, IL17A, IL6, and IFNG), six small molecules (Fe^2+^, NSC2921, sialate, CMP-N-glycolylneuraminate, N-glycolylneuraminate, CMP-N-acetylneuraminate), three cell processes (immune reactivity, B-cell activation, cell growth), and two diseases (metabolic disease and Duchenne muscular dystrophy). In addition, we identified four CMAH upstream-localized diseases (neoplasm, malaria, Mdx dystrophy, and nematode infections), three small molecules (NaCl, NADH, and ferrosulfate), eight functional classes (serotonin receptor, ncRNA, DNA-directed RNA polymerase, nuclease, PKC, electron carrier, oncogene, and muscin), and one protein (CYB5A). As shown in Figure 5B, the data set obtained from a representative potential signaling pathway accurately predicted previously reported metabolic disease models, such as human type II diabetes [21] and human Duchenne muscular dystrophy syndrome [16]. Taken together, this analysis suggests that regulation analysis of both Cmah and other proteins provide a valuable tool to evaluate human-specific diseases, caused by loss of CMAH function during human evolution.

## Discussion

Microarray analysis was selected to uncover the underlying molecular mechanisms necessary for animal-to-human xenotransplantation, as well as the metabolic conditions arising from the evolutionary loss of CMAH. The microarray expression data were validated by RT-qPCR analysis of selected genes from the lung, kidney, and heart of Cmah null mice. Overall, the decrease in expression of glycosyltransferases suggests a corresponding reduction in the cell surface sialylation and carbohydrate modification in the Cmah null mouse tissues. From the regulatory network identified from the Cmah null mice, we were able to show that many of the transcription factors and target genes associated with CMAH activity were linked in our network.

Recently, several studies have linked CMAH deactivation to hearing loss [15], skin healing delay [15], a human-like muscular dystrophy phenotype following combined mutation of the Dmd gene [16], abnormal B cell proliferation and antibody production, and type 2 diabetes-like syndromes [22]. To elucidate the role of CMAH in an animal model, a conventional knockout mouse has been constructed and made available to the scientific community [22]. We have also recently reported the first successful CMAH knockout in pigs [12]. First, we compared the expression profiles of Cmah^+/+^ and Cmah ^−/−^ mice littermates and between the Cmah null mouse and pig models. In this study, GalNT7 expression for Tn antigen in Cmah null mouse, unlike other family, significantly increased whereas, the expression of this gene in CMAH null pigs was significantly decreased. Moreover, ST6GalNac2 expression for Sialyl-Tn antigen and GalNT2, GalNT3, and GalNT4 expression for Tn antigen in CMAH null pigs were not changed compared to the control [12], whereas the expression of these genes in Cmah null mice was significantly decreased (Figure 2). While considerable differences in sialyltransferase expression between the Cmah null mouse and pig models were observed, their sialyltransferase expression profiles were strikingly similar overall. With respect to expression of H-D antigens, ST6GalNac3, ST6GalNac4, and ST6GalNac6 expression for sialyl-Tn antigen and GalNT6 expression for Tn antigen were all significantly down-regulated in both the Cmah null mouse and CMAH-null pig, suggesting a common expression signature for both models. This observation also suggests that the potential for Cmah null mice to serve as model for human-specific disease is becoming a reality, whereas that they have limitations because of their differences in gene expression and physiology compared to humans.

GO is an international standardized classification system for determining gene function, which supplies a set of controlled vocabulary to comprehensively describe the property of genes and gene products [23]. There are 3 ontologies in GO: cellular component, molecular function, and biological process [20]. In this study, analysis of molecular function showed that 204, 162, and 147 genes were assigned to 29 different functions based on gene background, while 42, 23, and 28 genes were involved in multiple functions in the lung, kidney, and heart, respectively (Files S1, S2, and S3). GO enrichment for gene background, based on the cellular component, showed that 34, 32, and 36 altered genes were mapped in the lung, kidney, and heart to GO terms in the database. As for the KEGG pathway analysis, it is worth noting that the following pathways were significantly (P value<0.0121528–0.0000001) enriched in the Cmah null mouse (Table 2): for the heart, focal adhesion; for the lung, retinol metabolism and glycerolipid metabolism; for the kidney, drug metabolism, arachidonic acid metabolism, complement and coagulation cascades, retinol metabolism, fatty acid metabolism, and circadian rhythm. The IPA further identified interacting modules involved in lipid metabolism and apoptosis signaling networks, which include fatty acids and their surrogate genes Pnlip (pancreatic lipase), Pnliprp1 (pancreatic lipase-related protein 1), and Pnliprp2 (pancreatic lipase-related protein 2). These significantly enriched pathways may imply that alterations in glycerolipid metabolism and fatty acid metabolism are involved in the pathogenesis of potentially fatal conditions, such as obesity, type II diabetes, and cancer. The results of the GO annotation and KEGG pathway analyses can therefore provide direction for future research. This study identified a common network in the three main organs, as the lung, kidney and heart, which are associated with lipid metabolism and small-molecule biochemistry.

The microarray analysis revealed distinct expression profiles for the lung, kidney, and heart from Cmah^+/+^ and Cmah ^−/−^ littermates. The microarray data were analyzed using the Ingenuity System Database software that includes the Ingenuity Knowledge Base (IKB) and the Global Molecular Network (GMN). These databases integrate published findings on biologically meaningful genetic or molecular gene/gene product interactions and identify functionally related gene networks [24]. The differential gene expression values (Cmah^+/+^ versus Cmah ^−/−^ litter mates) were entered into the IPA to determine the most highly regulated networks of gene interactions and to highlight the biological processes that are relevant to each of the treatments. Only networks with a score of 30 or higher were selected for further analysis. Networks describing the relationships between a subset of genes, their neighboring genes, and symbols representing the functional categories of the molecules are presented in Figure 4. Focus genes are denoted by red symbols for up-regulated genes and green symbols indicate down-regulated genes. Grey and open symbols are intermediate genes, placed in the network by the Ingenuity Software and shown in the literature to interact with genes in this dataset. Genes in gray have been shown to interact with the colored gene products that appear in this scheme.

The lung network (left of Figure 4) showed a score of 30 and contains 13 differentially regulated genes. Among them, 10 and 13 genes were involved lipid metabolism (P value: 3.21E-07–4.83E-02) and small-molecule biochemistry (3.21E-07–4.83E-02), respectively. The 5 genes (Cel, Lpl, Pnlip, Pnliprp1, and Pnliprp2) in this network have a central role in glycerolipid metabolism. These genes (Pnlip, Pnliprp1, and Pnliprp2) encode key lipolytic enzymes and are known to alter lipid metabolism. Pnliprp1, and Pnliprp2 encode two novel pancreatic lipase-related proteins, referred to as Pnlip-related proteins 1 and 2. Both these proteins have an amino acid sequence identity of 68% to Pnlip [25]. Pnliprp2 shows lipolytic activity that is marginally dependent on the presence of colipase, whereas the function of Pnliprp1 remains unclear [26]. Overall, these functionally related lipases play key roles in directing the regulation of fatty acid turnover and signaling. The most common symptoms associated with lipase deficiency are muscle spasms, acne, arthritis, gallbladder stress, formation of gallstones, bladder problems, and cystitis [27]. Therefore, our study has also provided the first evidence that a lower expression of Pnlip in Cmah null mice may be closely associated with above mentioned disease symptoms. The kidney network (middle of Figure 4), with the highest score (48), includes 18 genes. Among them, 10 and 13 genes were involved lipid metabolism (P value: 3.94E-087–4.82E-02) and small-molecule biochemistry (3.94E-08–4.82E-02), respectively. The heart network (right of Figure 4) received a score of 34 and contains 14 differentially regulated genes. Among them, 9 and 10 genes were involved lipid metabolism (P value: 1.14E-05–4.56E-02) and small-molecule biochemistry (1.14E-05–4.99E-02), respectively. Taken together, these data suggested that the gene interacting network predicted in the Cmah null tissues could be critical in understanding the role of evolutionary loss of CMAH function in human metabolism disorders.

PathwayAssist is a software application developed for navigation and analysis of biological pathways, gene regulation networks, and protein interaction maps [28]. Using Pathway Studio, we identified a number of protein interaction pathways with important roles following the evolutionary loss of CMAH function (Figure 5B). Comparing previously reported diseases caused by loss of CMAH function, we were able to accurately predict metabolic diseases, including human type II diabetes and human Duchenne muscular dystrophy syndrome, caused by CMAH inactivation. Therefore, our observations suggested that accuracy and efficiency in the interpretation of CMAH function is improved by Pathway Studio analysis.

Herein we identified 8 disease and disorders identified in the Cmah null tissues, based on the literature contained in the IPA knowledge base (P<0.05) (Table 3). Of them, specific disorders in Cmah null mice were closely associated with cardiovascular disease (Ptgds, Scd, and Egrl)-, skeletal and muscular disease (Adh1c, Dbp, Dkk3, Egr1, Herpud1, Scd, Snca, Ptgds, Timp4, Myl4, Alas2, and Fkbp5)-, and cancer (Cfd, Egr1, Scd, Timp4, Per2, Scgb1a1, Spon2, Slc46a3, and Gck)-associated genes in heart (Table 3). In conclusion, these findings suggest that CMAH inactivation could affect a variety of signaling pathways accompanying orchestrated gene expression changes. Therefore, our results argue that evolutionary loss of Neu5Gc affect complex regulation of cellular signaling pathways that involved in human diseases.

## Supporting Information

Figure S1 **Binding of natural xenoreactive antibodies in human sera to thymocytes from WT, Cmah-mKO (+/−; hetero) and Cmah-dKO (−/−; homo) mice.** Indirect immunofluorescence staining of thymocytes from WT, Cmah-mKO, and Cmah-dKO mice was used to detect xenoreactive antibody levels in healthy human serum samples (blood group: A, B, O, and AB). Detection of IgM or IgG binding was achieved by further incubating the cells with DyLight 649-conjugated Monkey anti-Human IgM or DyLight 488 Monkey anti-Human IgG Abs. Histogram profiles show differences in binding of natural antibodies present in human sera to the different thymocytes. Green lines indicate binding to thymocytes from WT; pink lines, binding to thymocytes from Cmah-mKO; sky blue lines, binding to thymocytes from Cmah-dKO, and gery lines indicate negative control WT thymocytes stained with secondary antibody alone.
(TIF)

Table S1 **Primer sets used for detection of sialyltransferases mRNA expression.**
(DOCX)

Table S2 **Primer sets for real-time RT-qPCR.**
(DOCX)

Table S3 **Common up- or down-regulated genes in lung, kidney, and heart of Cmah null mouse.**
(DOCX)

Table S4 **Networks predicted by Ingenuity Pathway Analysis in lung, kidney, and heart of Cmah-null mice.**
(DOCX)

File S1 **UP or down-regulated genes in lung of Cmah-null mice.**
(XLSX)

File S2 **UP or down-regulated genes in kidney of Cmah-null mice.**
(XLSX)

File S3 **UP or down-regulated genes in heart of Cmah-null mice.**
(XLSX)
