# Correction: Gene Expression and Pathway Analysis of Effects of the CMAH Deactivation on Mouse Lung, Kidney and Heart

**DOI:** 10.1371/journal.pone.0118915

**Published:** 2015-03-05

**Authors:** The PLOS ONE Staff

There is an error in the Funding statement. Please view the correct Funding statement here.

This work was supported by academic research funds of Konkuk University in 2013, Seoul, South Korea. The funders had no role in study design, data collection and analysis, decision to publish, or preparation of the manuscript.
