# Supplementary figures and images for: Gene Expression and Pathway Analysis of Effects of the CMAH Deactivation on Mouse Lung, Kidney and Heart
Source: PLoS One. 2014 Sep 17;9(9):e107559. doi: 10.1371/journal.pone.0107559 (PMC4167996; doi:10.1371/journal.pone.0107559)

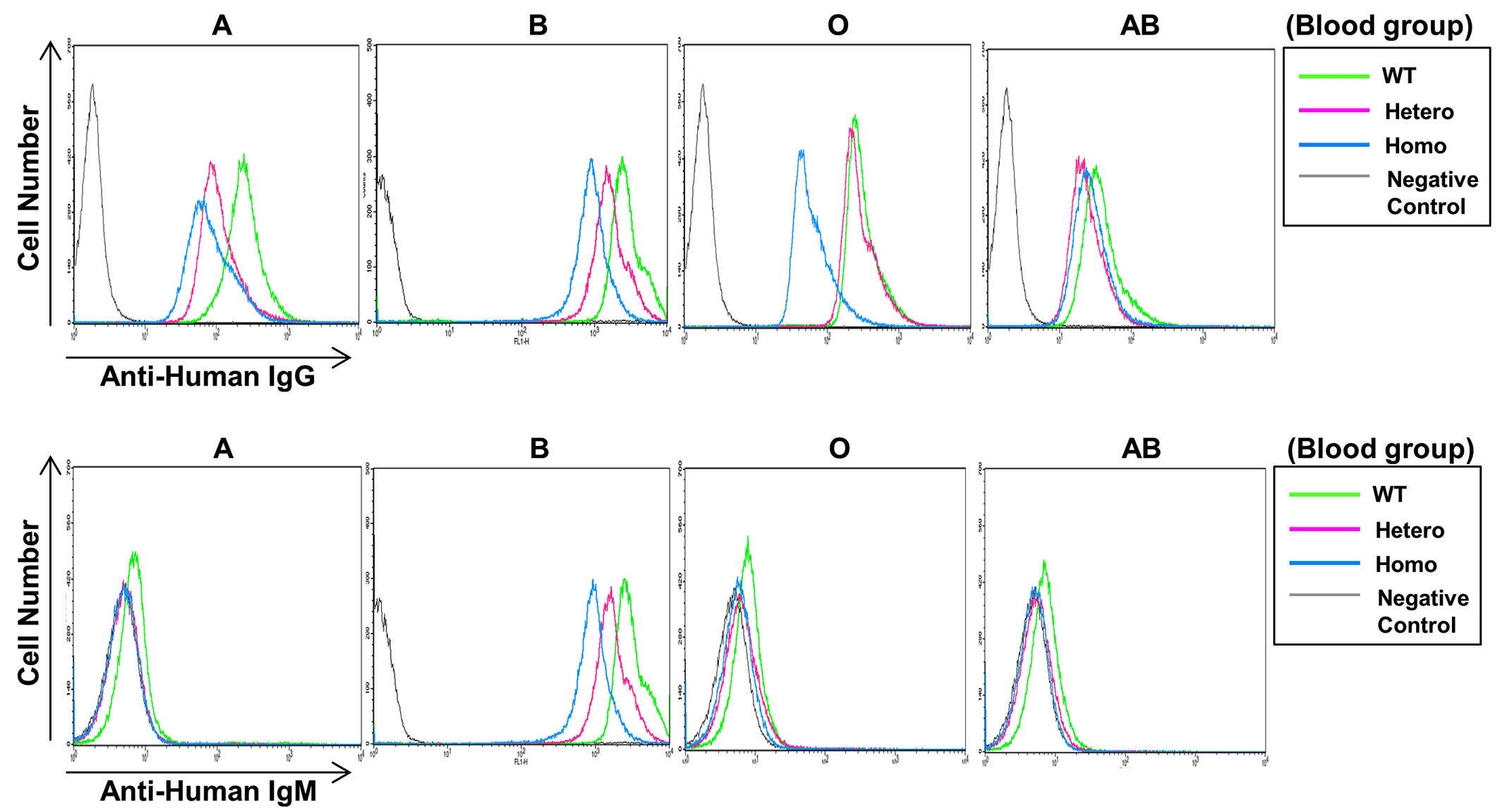

Supplement: Figure S1 — Binding of natural xenoreactive antibodies in human sera to thymocytes from WT, Cmah-mKO (+/−; hetero) and Cmah-dKO (−/−; homo) mice. Indirect immunofluorescence staining of thymocytes from WT, Cmah-mKO, and Cmah-dKO mice was used to detect xenoreactive antibody levels in healthy human serum samples (blood group: A, B, O, and AB). Detection of IgM or IgG binding was achieved by further incubating the cells with DyLight 649-conjugated Monkey anti-Human IgM or DyLight 488 Monkey anti-Human IgG Abs. Histogram profiles show differences in binding of natural antibodies present in human sera to the different thymocytes. Green lines indicate binding to thymocytes from WT; pink lines, binding to thymocytes from Cmah-mKO; sky blue lines, binding to thymocytes from Cmah-dKO, and gery lines indicate negative control WT thymocytes stained with secondary antibody alone. (TIF) [file pone.0107559.s001.tif]
