# Supplementary material for: Gene Expression and Pathway Analysis of Effects of the CMAH Deactivation on Mouse Lung, Kidney and Heart
Source: PLoS One. 2014 Sep 17;9(9):e107559. doi: 10.1371/journal.pone.0107559 (PMC4167996; doi:10.1371/journal.pone.0107559)
Supplement: Table S1 — Primer sets used for detection of sialyltransferases mRNA expression. (DOCX) [file pone.0107559.s002.docx]

**Table S1. Primer sets used for detection of sialyltransferases mRNA expression**

| Antigen | Gene | Forward primer | | Reverse primer | Product(bp) | |
| --- | --- | --- | --- | --- | --- | --- |
| H-D antigen | ST6Gal1 | | TGTGGGCACAAAAACTACCA | CTGGGGCTTGAGGATGTAAA | | 232 |
|  | ST3Gal1 | | GACAGTCCACAACGCTCTGA | CCCATACGAGGAGTCCTTCA | | 214 |
|  | ST3Gal2 | | CCCTGCTCTTCACCTACTCG | GTCCAGACGGGTGAGATGTT | | 232 |
|  | ST3Gal3 | | CTCCAATGGGAAGACTCCAA | TCATCATGGCTGAAGCGTAG | | 187 |
|  | ST3Gal4 | | CGATGGACTTCCACTGGATT | GCAGAGGTGTAGAGCCAAGG | | 239 |
|  | ST3Gal5 | | TCAAGTGGCTTCAAGCAATG | GTAGCCAAGACAACGGCAAT | | 238 |
|  | ST3Gal6 | | GCCCTTTCAAAACTGCAGAG | TCCCAACTTCCTCTTCATGG | | 187 |
| Sialy-Tn antigen | ST6GalNac2 | | TGCCTACGGATTCATCACAAA | CGTTGGTACAGCCAAAGGAT | | 159 |
|  | ST6GalNac3 | | TGTAACCACTGTGCCATCGT | ATGTGACACAACTCGGACCA | | 162 |
|  | ST6GalNac4 | | ATCAGGCTCCTTTCTCAGCA | CGGCCCTTCTCAAAGTAGTG | | 150 |
|  | ST6GalNac6 | | ACGCCGGAGAGAGATGAGTA | CGCTGGAGAAACTCCACTTC | | 182 |
| Forssman antigen | GBGT1 | | CCCTTCAGGAAGGAGAAACC | CGCTGAATGAAGCAGGTGTA | | 230 |
| Tn antigen | GalNT2 | | CCCTTCAAGTGGTACCTGGA | TGCTTCACCGACTTCTCCTT | | 203 |
|  | GalNT3 | | CTTCTGGCAAGCCGTTTAAG | GTGGACCATGCTTCATTGTG | | 232 |
|  | GalNT4 | | ATAACGCTCCCGACAACAAC | TGAGGGTGAAAAATCGTTCC | | 250 |
|  | GalNT6 | | GCGTCTTCATGCTCTTCCTC | AGCTCAGCAGGGGTGTAGAA | | 244 |
|  | GalNT7 | | TCTTACGCAGTTTGCTGGTG | TTCAACATGAGGCCATGGTA | | 206 |
