# Supplementary material for: Gene Expression and Pathway Analysis of Effects of the CMAH Deactivation on Mouse Lung, Kidney and Heart
Source: PLoS One. 2014 Sep 17;9(9):e107559. doi: 10.1371/journal.pone.0107559 (PMC4167996; doi:10.1371/journal.pone.0107559)
Supplement: Table S2 — Primer sets for real-time RT-qPCR. (DOCX) [file pone.0107559.s003.docx]

**Table S2. Primer sets for real-time RT-qPCR**

| Group | Genes | Forward primer | Reverse primer | Product(bp) |
| --- | --- | --- | --- | --- |
| Glycerolipid metabolism | Pnlip | AGCCATTGGAAGGATCACAG | CGTCGATGTCAACAATTTGG | 248 |
|  | Cel | GACCCTTGGGTTTCCTTAGC | TTGTATGGGGAGAGGGTCTG | 190 |
|  | Pnliprp1 | ATCAACACTCGCTTCCTGCT | TCAACCACCCAGTTCTCCTC | 167 |
|  | Dgat2 | TCCAGCTGGTGAAGACACAC | GATGCCTCCAGACATCAGGT | 215 |
| Fatty acid metabolism | Cyp4a12b | GCCTTCATCACAACCCAACT | GGTATGGGGATTGGGACTCT | 226 |
|  | Adh1 | ACAAACCCTTCACCATCGAG | CCTTCTCCAACGCTCTCAAC | 184 |
|  | Cyp4a14 | TGGGGAGATCAGATCCAAAG | GACAGAGTCCGCCATGATTT | 173 |
| Regulation of actin cytoskeleton | Actb | AGCCATGTACGTAGCCATCC | CTCTCAGCTGTGGTGGTGAA | 228 |
|  | Myl7 | TCAGCTGCATTGACCAGAAC | CCCGAAGAGTGTGAGGAAGA | 164 |
|  | Itgb6 | TCTGAGGATGGAGTGCTGTG | GGCACCAATGGCTTTACACT | 210 |
|  | Pik3r1 | CCCAAGCTGGATGTGAAGTT | TGCTTCGATAGCCGTTCTTT | 207 |
