# Supplementary material for: Gene Expression and Pathway Analysis of Effects of the CMAH Deactivation on Mouse Lung, Kidney and Heart
Source: PLoS One. 2014 Sep 17;9(9):e107559. doi: 10.1371/journal.pone.0107559 (PMC4167996; doi:10.1371/journal.pone.0107559)
Supplement: Table S3 — Common up- or down-regulated genes in lung, kidney, and heart of Cmah null mouse. (DOCX) [file pone.0107559.s004.docx]

**Table S3. Common up- or down-regulated genes in lung, kidney, and heart of Cmah-null mouse**

| **Up-regulated genes** | |  |  |  |
| --- | --- | --- | --- | --- |
| **Genes** | **Definition** | **Fold change** | | |
|  |  | **Lumg** | **Kidney** | **Heart** |
| Entpd4 | ectonucleoside triphosphate diphosphohydrolase 4 | 1.795 | 2.121 | 1.653 |
| Bst2 | bone marrow stromal cell antigen 2 | 2.201 | 1.909 | 1.166 |
| Ddx3y | DEAD (Asp-Glu-Ala-Asp) box polypeptide 3, Y-linked | 1.604 | 1.902 | 3.010 |
| Stat1 | signal transducer and activator of transcription 1 | 1.550 | 1.555 | 1.047 |
| Cdkal1 | CDK5 regulatory subunit associated protein 1-like 1 | 1.634 | 1.365 | 1.562 |
| Igtp | interferon gamma induced GTPase | 1.281 | 2.184 | 1.588 |
| Iigp2 | interferon inducible GTPase 2 | 1.426 | 1.835 | 1.544 |
| Adh1 | alcohol dehydrogenase 1 (class I) | -1.039 | 1.819 | 1.872 |
| Inmt | indolethylamine N-methyltransferase | -1.014 | 1.536 | 2.171 |
| Fkbp5 | FK506 binding protein 5 | -1.937 | 1.529 | 2.051 |
| Per5 | period homolog 2 (Drosophila) | -1.174 | 1.515 | 3.474 |
| **Down-regulated genes** | |  |  |  |
| **Genes** | **Definition** | **Fold change** | | |
|  |  | **Lumg** | **Kidney** | **Heart** |
| Per1 | period homolog 1 (Drosophila) | -1.525 | -1.577 | 1.158 |
| Nrld1 | nuclear receptor subfamily 1, groupD, member 1 | -2.263 | -1.661 | -1.528 |
| Dbp | D site albumin promoter binding protein | -4.220 | -1.665 | 2.742 |
| Sybl1 | synaptobrevin like 1 | -2.317 | -1.962 | -1.609 |
| LOC100047427 | similar to thyroid hormone receptor | -2.950 | -2.016 | -1.744 |
| Cfd | complement factor D (adipsin) | -2.674 | -3.009 | -4.449 |
| Irs2 | insulin receptor substrate 2 | -1.267 | -1.502 | -1.867 |
| Wnk1 | WNK lysine deficient protein kinase 1 | -1.049 | -1.658 | -1.640 |
| Chka | choline kinase alpha (Chka), transcript variant 2 | 1.190 | -1.882 | -1.803 |
| Gm129 | gene model 129, (NCBI) | -1.597 | -1.598 | 1.927 |
