# Supplementary material for: Gene Expression and Pathway Analysis of Effects of the CMAH Deactivation on Mouse Lung, Kidney and Heart
Source: PLoS One. 2014 Sep 17;9(9):e107559. doi: 10.1371/journal.pone.0107559 (PMC4167996; doi:10.1371/journal.pone.0107559)
Supplement: Table S4 — Networks predicted by Ingenuity Pathway Analysis in lung, kidney, and heart of Cmah-null mice. (DOCX) [file pone.0107559.s005.docx]

**Table S4. Networks predicted by Ingenuity Pathway Analysis in lung, kidney, and heart of Cmah-null mice**

| **Tissue** | **Associated Network Functions** | **Score** | **Focus molecules** |
| --- | --- | --- | --- |
| Lung | Lipid metabolism, Small molecule biochemistry, Vitamin and mineral metabolism | 30 | 13 |
|  | Antimicrobial response, Dermatological disease and conditions, Cellular compromise | 27 | 12 |
|  | Cell death and survival, Connective tissue development and function, Cell-to-cell signaling and interaction | 19 | 9 |
| Kidney | Lipid metabolism, Small molecule biochemistry, Vitamin and mineral metabolism | 48 | 18 |
|  | Endocrine system development and function, Small molecule biochemistry, Drug metabolism | 26 | 11 |
|  | Cell cycle, Cell death and survival, Hematological syatem development and function | 2 | 1 |
| Heart | Lipid metabolism, Small molecule biochemistry, Vitamin and mineral metabolism | 34 | 14 |
|  | Organ metabolism, Skeletal and muscular system development and function, Cellular development | 31 | 13 |
|  | Endocrine system development and function, Small molecule biochemistry, Drug metabolism | 2 | 1 |
